# Tensile Strength of Nylon Sutures Versus Polyglycolic Acid Sutures Immersed in Camu camu (*Myrciaria dubia*) and Tumbo (*Passiflora tripartita*) Juices: A Linear Regression Model

**DOI:** 10.1155/ijod/4015454

**Published:** 2026-02-04

**Authors:** Tania Mamani-Salinas, Daniel Alvitez-Temoche, Cesar Mauricio-Vilchez, Fran Espinoza-Carhuancho, Oscar Sotomayor, Julia Medina, Frank Mayta-Tovalino

**Affiliations:** ^1^ Faculty of Dentistry, Research, Innovation and Entrepreneurship Unit, Universidad Nacional Federico Villarreal, Lima, Peru, unfv.edu.pe; ^2^ Bibliometrics Evidence Evaluation and Systematic Reviews Group (BEERS) Human Medicine Career, Universidad Científica del Sur, Lima, Peru, cientifica.edu.pe; ^3^ EVIDENTIA Research Group, Universidad Nacional Mayor de San Marcos, Lima, Peru, unmsm.edu.pe; ^4^ Vicerrectorado de Investigación, Universidad San Ignacio de Loyola, Lima, Peru, usil.edu.pe

**Keywords:** exotic fruit, in vitro, nylon, tensile strength

## Abstract

**Objective:**

To evaluate the predictors of tensile strength of nylon and polyglycolic acid when immersed in Peruvian camu camu and tumbo juices.

**Methods:**

An in vitro experimental and comparative study was conducted following the CRIS guidelines for reporting laboratory studies. A total of 192 samples of sutures were tested under laboratory conditions, measuring their initial resistance at days 3, 7, and 14. The juices, prepared in an artisanal way to preserve their natural characteristics, were monitored for pH daily. Mechanical tests were performed using a digital tensile machine, and data were analyzed with Stata 17.0 statistical software using Student’s *t*‐tests and linear regression.

**Results:**

Both materials displayed a timewise decrease in strength, with polyglycolic acid being stronger than nylon at all time points measured. At time 0 polyglycolic acid and nylon were noted as 12.23 ± 0.40 N and 8.94 ± 0.17 N, respectively. At day 14 polyglycolic acid and nylon were noted as 11.24 ± 0.38 N and 7.59 ± 0.38 N, respectively. The camu camu juice and the tumbo juice displayed similar effects (*p* > 0.05), and pH was the only feature to display a significant decrease per unit variation (*p* < 0.05).

**Conclusion:**

Such a conclusion tends to confirm the greater acid resistance of polyglycolic acid over nylon and adds more weight to pH as a variable affecting suture degradation. We may learn to make more intelligent choices of absorbable sutures at the operating table.

## 1. Introduction

The use of materials to assist in the closure of wounds goes back to Egyptian scrolls from 3500 bc. The techniques and materials used in the surgery were very different from those used today, primarily utilizing naturally occurring materials: leather bands, tree bark fiber, animal tendons and using human hair [1]. The healing of surgical wounds is a complex process involving the generation of an extracellular matrix capable of binding the edges of the lesion and providing structural support to cells, while simultaneously promoting the regeneration of blood vessels and restoring the degree of functional resistance of the tissue to mechanical forces [2–4].

Suturing remains today a sine qua non of oral surgery, a material without which surgical procedures in the oral cavity will not succeed: just take a look at the role of sutures in holding together tissues during the process of healing. Their judicious choice will weigh very heavily on the course of events clinically and thus on the patient’s lot. An understanding of the nature of suture threads, of tensile strength especially, is vital to the choice of materials and prevention of complications, improved results, the patient’s safety, and ease in the conduct of everyday surgery in the clinic [5].

The sutures used in the cavity of the mouth have some peculiarities that result from the constant presence of saliva and the functions it performs, such as mastication, speech, deglutition, etc.; hence, for each kind of oral surgery, a certain kind of suture thread must be chosen that must possess well‐determined physical characteristics and properties [3]. The suture threads intended for procedures in the oral cavity must comply with a series of physical and chemical characteristics that minimize the risk of damage to the oral mucosa. These properties include tensile strength, dimensional stability, memory, knot security, and flexibility. In addition, subjective criteria such as the maneuverability of the material and the surgeon’s personal preferences are involved, which also influence the choice of the most appropriate suture thread for each case [4].

The tensile strength is defined as the greatest force before rupture in the material occurs. The gradual waste and retention of 70–80%. of the original power is a measure of mechanical effect and recovery, but not of tensile strength [6].

Acid juices and drinks have a graver effect on the tensile quality of the threads; when in contact with a solution of lower acidity, at least quickly, the fibers begin to lose their strength and are wont to yield before healing, more easily rendering the parts that they should hold together incapable of surgical union. The tensile strength, in this new sense and meaning of the word, safeguards from every form of failure, and its endurance depends not only on the chemical constitution of the medium with which it is associated but also on the nature of the suture itself, the time of contact, and the rough, devious course through the parts that it traverses. The discussion of tensile strength should go beyond its basic definition to include the multiple factors that influence this property in suture materials. In particular, the effects of pH and acidic environments are critical, as they can accelerate polymer degradation, weaken fiber integrity, and reduce the ability of sutures to maintain tissue approximation over time [3–8].

Therefore, the aim of this study was to evaluate the predictors of the tensile strength of nylon and polyglycolic acid when immersed in the Peruvian juices of camu camu (*Myrciaria dubia*) and tumbo (*Passiflora tripartita*).

## 2. Materials and Methods

### 2.1. Ethics

The present study was approved by the Research Ethics Committee of the Faculty of Dentistry of the Universidad Nacional Federico Villarreal under the code of act Number 096‐05‐2024. It should be noted that, due to the in vitro nature of the study and the fact that it did not involve the participation of human beings, it was not necessary to apply the ethical principles related to research involving human subjects, nor was it necessary to require informed consent.

### 2.2. Study Design

An experimental, in vitro, prospective, longitudinal, and comparative study was conducted. The CRIS Guidelines (checklist for reporting in vitro studies) were used for data reporting [7].

### 2.3. Sample Size

To calculate the sample, the data from our pilot study were used (mean and standard deviation). Using the mean comparison formula, with an α = 0.05, β = 0.8, the mean of the first group was 0.12, mean of the second group was 0.79, standard deviation of the first group was 0.25 and the standard deviation of the second group was 0.1. The result yielded 4 samples per group; however, it was decided to increase to an average of *n* = 12 per group. Making a total of *N* = 192 specimens. Finally, all analysis was performed using Stata 17.0 software.

### 2.4. Selection Criteria

The selection criteria were carefully established to ensure the quality and consistency of the materials used. Only 5/0‐gauge nylon and polyglycolic acid suture threads of a single brand were included, ensuring that they were sealed and in perfect condition. On the other hand, suture threads of other calibers, those that were expired, or those whose packaging was open or damaged were excluded. This rigorous process allowed us to maintain the integrity and reliability of the materials used in the study.

### 2.5. Allocation

**Figure 1:**
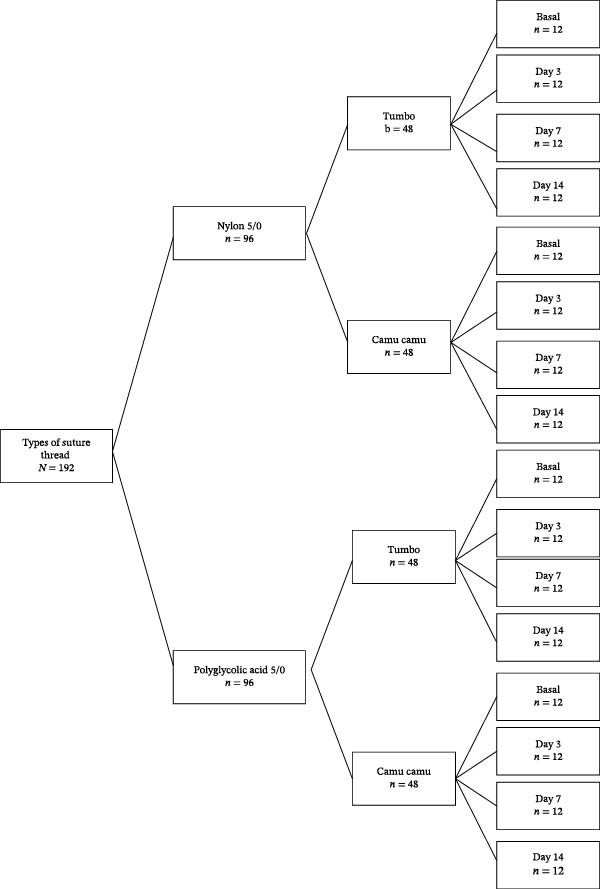
Group distribution diagram.

To obtain the samples, the distribution of the groups was first defined. The selected samples were classified into two groups (Nylon 5/0 and polyglycolic acid); each group was confronted with two subgroups of native Peruvian fruit juices (tumbo and camu camu) (Figure 1).

### 2.6. Obtaining Sutures

For the present study we have selected the suture threads from the Peruvian brand Tagum Tagummedica, Lima‐Peru, based on their technical characteristics and proven reliability. The selected nylon sutures were black, monofilament sutures of 5/0 gauge with a 15 mm needle in a 3/8 circle configuration, with a strand length of 75 cm, identified by code NN0848.G. The polyglycolic acid sutures (brand Cirugía Peruana Plus, Lima‐Peru) were also 5/0 gauge, with a 20 mm needle in a 3/8 circle, and a 70 cm strand length, identified by code GS0824.J. Laboratory specifications and pilot testing showed that specimens should ideally have a minimum length of 45 mm for a correct measurement of their tensile properties. Therefore, we excised one specimen from every suture thread and multiplied by three the number of boxes of each type so as to get sufficient units for the rest of our analysis: 36 in all.

### 2.7. Preparation of Fruit Juices

**Figure 2:**
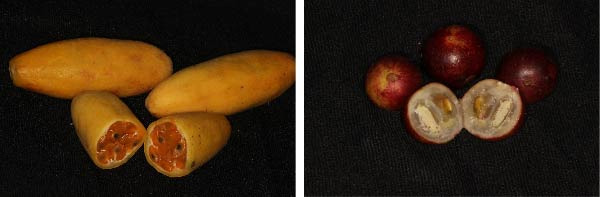
Native Peruvian fruits such as Passiflora tripartita “Tumbo” (yellow) and Myrciaria dubia “Camu camu” (red).

The preparation of the juices was carried out following specific procedures to preserve the properties of the fruits. The camu camu, originally from the Peruvian Amazon, was washed with distilled water, dried with absorbent paper, and its pulp was removed to liquefy it without adding water, avoiding altering its natural acidity. Subsequently, the obtained extract was filtered using 90 mm nylon paper (Elicrom brand) to eliminate pulp remains and obtain pure juice. Similarly, Tumbo, a fruit from the Peruvian highlands, was washed, dried, and split to extract its pulp. This was liquefied without adding water and filtered under the same conditions as camu camu. Once obtained, both juices were stored in a refrigerator at 21°C to avoid decomposition and maintain their properties intact during the study (Figure 2).

### 2.8. Specimen Preparation

The samples were prepared in the dental operatory laboratory of the Faculty of Dentistry of the Universidad Nacional Federico Villarreal. Sixteen sterile flasks were labeled to identify each group, and the needles of the suture threads were cut to facilitate the manipulation of the specimens. Twelve specimens were placed in each bottle, and 80‐ml of camu camu or tumbo juice was added, as indicated on the label. Four jars of the initial group did not contain juice, as their specimens were evaluated directly in the tensile machine. The jars were sealed and stored in a refrigerator at 21°C to slow the decomposition of the juices. Daily, the pH was monitored using a pocket meter (Hanna Instruments model HI98103), ensuring that it remained within the range of 2.7–3.5. In case of deviation, the juice was replaced with freshly prepared juice to ensure constancy of experimental conditions. In the present study, the control group was set by the polyglycolic acid sutures, which were also immersed in fruit juices and were treated in the same way as the other groups; specimens followed the same preparation, storage, and monitoring protocol; they were immersed in camu camu or tumbo juice, with daily control of pH and tensile strength assessment at days 3, 7, and 14.

### 2.9. Tensile Test

**Figure 3:**
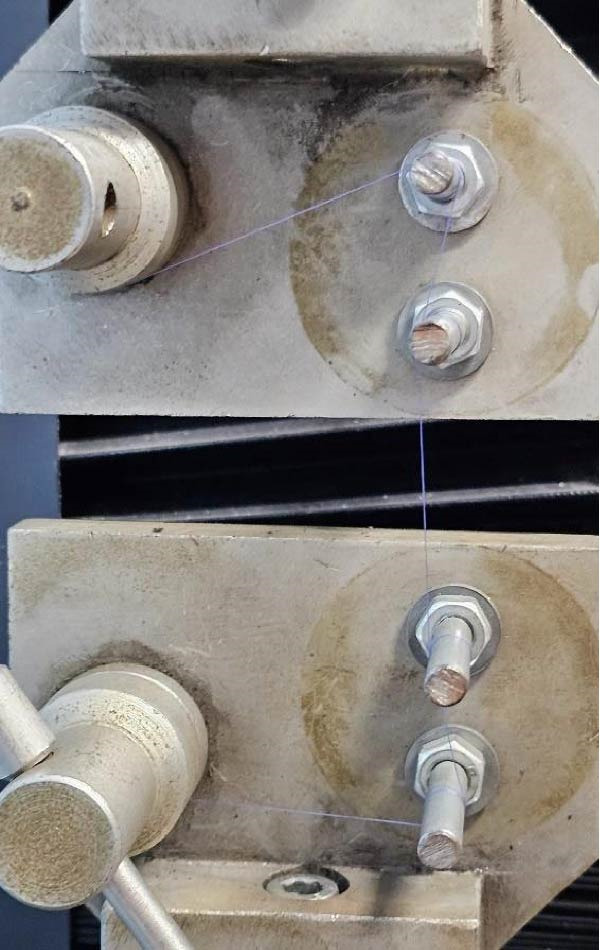
Tensile strength essay.

Samples were transported in a cooler containing a Tippic Industries Gel Pack, designed to hold low temperatures within the thermal packaging, keeping stable conditions within 21°C during transport to the High Technology Laboratory. At the High Technology Laboratory, each sample vial was rinsed with sterile water, and the samples were ventilated and dried on absorbent paper. Once dry, each sample was prepared by individually tying it in a knot around two metal poles secured on a digital Vernier mechanical testing machine (model LG CMT ‐ 5 L) with a fixed distance of 15 mm and a tensile rate of 25 cm/min. Each sample was drawn until the point of failure, documenting the maximum load in newtons (N) in the datalogging sheet. This procedure was repeated during intervals during the prescribed time periods, baseline measurement, days 3, 7, and 14 of the experiment, and the evaluation of the mechanical characteristics of the samples was conducted in a consistent manner (Figure 3).

### 2.10. Analysis Plan

The data obtained were analyzed descriptively, being presented in tables containing means, standard deviations, maximums, and minimums for each group of suture threads, which constituted a group characteristic that served to seek for statistically significant differences between the studied groups. The distribution was assessed by means of the Shapiro–Wilk normality test. Once normality was confirmed, Student’s *t*‐test for independent samples was applied, comparing the tensile strength between the experimental groups. And a linear regression model was applied to evaluate the influence of the time of immersion and the tensile strength shown by the behavior of the materials in the different periods evaluated. The first processing of the data was carried out in a matrix created in Microsoft Excel—so that the information is adequately structured—and later the data were analyzed with statistical software Stata version 17.0, where the detailed analyses were carried out.

## 3. Results

**Figure 4:**
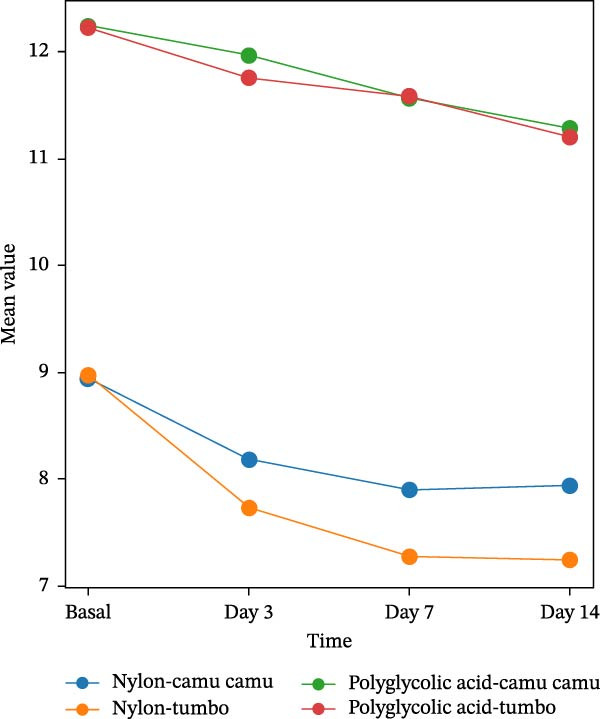
Tensile strength in nylon and polyglycolic acid sutures exposed to camu camu and tumbo.

The tensile strength of the nylon and polyglycolic acid yarns always showed a continuous decrease over time. In the basal analysis, the values for nylon were 8.94 ± 0.17 N in camu camu juice and 8.97 ± 0.41 N in tumbo juice; the polyglycolic acid values for strength were higher, at 12.23 ± 0.40 N and 12.21 ± 0.18 N, respectively. Substantial reductions in strength values were noted on the third day, but especially for nylon submerged in the tumbo juice (7.73 ± 0.23 N). On the seventh day, the nylon in tumbo showed a lower resistance value (7.28 ± 0.10 N) than any other materials reported. On day 14 both materials continued to reflect a downward trend, although polyglycolic acid maintained a superior strength overall. The differences between camu camu and tumbo juices were not statistically different (*p*  > 0.05), indicating the effect of both media on yarn degradation was similar (Table 1) (Figure 4).

**Table 1:** Tensile strength of nylon versus polyglycolic acid at four times (baseline, 3rd, 7th, and 14th day).

Time	Nylon		Polyglycolic acid		*p* ^∗^
	Camu camu	Tumbo	Camu camu	Tumbo	
Baseline measurement	8.94 ± 0.17	8.97 ± 0.41	12.23 ± 0.40	12.21 ± 0.18	>0.05
Day 3	8.18 ± 0.28	7.73 ± 0.23	11.96 ± 0.18	11.75 ± 0.31	>0.05
Day 7	7.89 ± 0.22	7.28 ± 0.10	11.56 ± 0.23	11.57 ± 0.42	>0.05
Day 14	7.94 ± 0.08	7.24 ± 0.19	11.28 ± 0.42	11.20 ± 0.34	>0.05

*Note:* Values were expressed in N (Newtons). *p* ^∗^: Shapiro–Wilk normality test.

The data indicated a noticeable reduction in the tensile strength of both nylon and polyglycolic acid yarns during the four times measured (basal, days 3, 7, and 14). Nylon had an initial average resistance of 8.9 ± 0.31 N, while polyglycolic acid measured 12.22 ± 0.30 N. At day 3, both materials had reduced to 7.95 ± 0.34 N and 11.86 ± 0.27 N, respectively. By day 7, nylon had reduced to 7.59 ± 0.35 N and polyglycolic acid had reduced to 11.57 ± 0.33 N. Finally, by day 14, the resistance values of nylon were 7.59 ± 0.38 N and polyglycolic acid was 11.24 ± 0.38 N. The difference between the two materials was statistically significant at every time measure (*p*  < 0.05); demonstrating that polyglycolic acid was more resistant than nylon throughout every measurement (Table 2).

**Table 2:** Comparison of the tensile strength values of suture threads at different times.

Day	Group	Mean	Standard deviation	95% confidence intervals		*p* ^∗∗^
Baseline measurement	Nylon	8.90	0.31	8.82	9.09	<0.05
	Polyglycolic acid	12.20	0.30	12.09	12.35	
Day 3	Nylon	7.95	0.34	7.81	8.10	<0.05
	Polyglycolic acid	11.80	0.27	11.74	11.97	
Day 7	Nylon	7.59	0.35	7.44	7.73	<0.05
	Polyglycolic acid	11.50	0.33	11.42	11.71	
Day 14	Nylon	7.50	0.38	7.43	7.76	<0.05
	Polyglycolic acid	11.20	0.38	11.08	11.40	

*Note:* *p* ^∗∗^, student’s *t*‐test.

The tensile strength values of the yarns immersed in the tumbo and camu camu juices showed similar behavior throughout the time evaluated. At basal time, the average values were 10.59 ± 1.68 N for Tumbo and 10.58 ± 1.71 N for Camu camu, with no significant differences between the two (*p* = 0.98). As time progressed, progressive decreases in resistance were observed, with values of 9.22 ± 2.03 N for Tumbo and 9.61 ± 1.72 N for Camu camu on day 14. However, these differences were also not significant (*p*  > 0.05), indicating that both juices had a comparable impact on the mechanical properties of the yarns evaluated (Table 3).

**Table 3:** Tensile strength values of suture threads immersed in different fruit juices.

Day	Group	Mean	Standard deviation	95% confidence intervals		*p* ^∗∗^
Baseline measurement	Tumbo	10.59	1.68	9.88	11.30	0.98
	Camu camu	10.58	1.71	9.86	11.30	
Day 3	Tumbo	9.74	2.07	8.86	10.61	0.57
	Camu camu	10.07	1.94	9.25	10.89	
Day7	Tumbo	9.43	2.21	8.49	10.36	0.61
	Camu camu	9.72	1.88	8.93	10.52	
Day14	Tumbo	9.22	2.03	8.36	10.08	0.47
	Camu camu	9.61	1.72	8.88	10.34	

*Note:* *p* ^∗∗^, student’s *t*‐test.

For every unit variation in the type of suture, the mean tensile strength decreased 0.53 units (95% CI: −2.12 to 1.05); however, this was not statistically significant (*p*  > 0.05). For each unit of variation in the type of fruit used, the mean tensile strength increased 0.93 units (95% CI: 0.32–1.53) and was significant (*p*  < 0.05). Overall, compared with the baseline measurement, the mean strength of all the subjects increased 0.23 units (95% CI: −0.00 to 0.47) and was not statistically significant (*p*  > 0.05). The average resistance from day 0 to day 3 increased 0.36 units (95% CI: 0.05–0.68), which was significant (*p*  < 0.05). By day 7, the average tensile strength increased 0.52 units (95% CI: 0.26–0.79) and was also significant (*p*  < 0.05). Of interest, for each unit variation in pH, the mean tensile strength decreased 3.22 units (95% CI: −5.53 to −0.90), which was statistically significant (*p*  < 0.05) (Table 4).

**Table 4:** Linear regression of the tensile strength of suture threads immersed in two fruit juices in four stages.

Variables	Tensile strength (Day 14)			
	β Crude			
	Coefficient			CI 95%
Ref. Polyglycolic acid Suture type	−0.53	0.504		−2.12a 1.05
Ref. Camu camu Fruit type	0.93	0.003		0.32a 1.53
Basal	0.23	0.059		−0.00a 0.47
Day 3	0.36	0.023		0.05a 0.68
Day 7	0.52	0.000		0.26a 0.79
pH	−3.22	0.008		−5.53a −0.90

## 4. Discussion

Camu camu (*Myrciaria dubia*) or Tumbo (*Passiflora tripartita*) juices were selected since these are “relatively” very acidic and have a low pH. These fruits are known to be rich in organic acids and potentially bioactive compounds, which could reproduce the acidic features of the oral cavity associated with certain unhealthy dietary habits, metabolism by bacteria, or reduced buffering ability of saliva. These “clinically” acceptable conditions predetermine the factors that favor the destruction of suture materials, thereby reducing the acceptability of their ultimate or remaining tensile characteristics as well as the capacity to withstand a given volume dose of the suture materials. The immersion of the absorbable and non‐absorbable sutures in such juices could predict the behavior of the materials under acidic stress and help the clinician to choose the appropriate sutures for oral and periodontal surgeries [8–12].

In periodontal surgery, the adequate choice of suture technique, surgical needle, thread diameter, and the appropriate type of thread are fundamental to achieving optimal healing by first intention, avoiding suture dehiscence, future scarring, and infections. In this sense, the present research aimed to evaluate one of the most relevant mechanical properties of suture threads used in surgical procedures: tensile strength. For this purpose, two types of sutures used in periodontal surgery were selected: nylon, a non‐absorbable material, and polyglycolic acid, an absorbable material. Both were exposed to the action of two juices from fruits native to the region (camu camu and tumbo) known for their acidic nature, with pH values of 3.3 and 2.8, respectively. The objective of utilizing these media is to find whether the acidity of these liquids influences the mechanical behavior of the yarns and their power of resisting traction at the different times (basal, days 3, 7, and 14), which correspond to the usual periods of removal from the oral cavity of the sutures used in periodontal surgery.

All the results were statistically analyzed and showed that there was a difference in the tensile strength of nylon and polyglycolic acid and that polyglycolic acid had a greater strength, having an average score of 12.23 N before the test of loading compared to the nylon average score of 8.97 N. Student’s *t*‐test was applied and showed a significant difference between the kinds of suture thread on the four occasions mentioned. The results were accompanied by a value of *p*  < 0.05. So, from the findings, noting that tensile strength decreased progressively over the four occasions means the length of time the suture thread is exposed to an aggressive medium is an important factor affecting the degradation of the suture threads. This is important because there should be consideration of immersion time (or exposure to the organism) when evaluating the durability and efficacy of sufficient suture materials.

In the study by Chu and Moncrief [8], they mention that the pH of the medium is another important factor influencing the performance of absorbable sutures more than that of non‐absorbable sutures, and that a strong alkaline condition would have a more adverse effect on the strength of suture materials than physiological and acidic pHs. The results obtained in this investigation agree with similar studies by Saravanakumar et al. [9] and Abellán et al. [10], and Khiste et al. [4] agree on the mechanical superiority of polyglycolic acid, a case also supported by Manfredini et al. [11] when comparing different calibers in the artificial saliva. For their part, Abullais et al. [12] highlight the influence of extrinsic factors such as diet, hygiene, tobacco consumption, or medication on the degradation of the thread in the oral environment.

On the other hand, in the study by Saravanakumar et al. [9], they evaluated the tensile strength of absorbable and non‐absorbable sutures, concluding that polyglycolic acid had minimal deterioration and catgut 5/0 showed maximum deterioration of tensile strength, while, among the non‐absorbable sutures, silk 4/0 showed maximum deterioration of tensile strength and polypropylene 3/0 showed minimal deterioration. In the research conducted by Abellan et al. [10], they mentioned that exposing suture threads to different pH media does not affect the integrity of the suture material. They also mention that, of the 5 types of suture threads, polyglycolic acid and silk showed the highest strength values, but that polyglycolic acid was the suture thread that showed the highest tensile strength value under natural conditions and that it had a significant decrease after remaining for 5–7 days.

In research conducted by Khiste et al. [4], there was a comparison of three types of suture threads: polyglycolic acid, polyglactin, and poly (glycolide‐co‐є‐caprolactone). The authors reported higher tensile strength for polyglycolic acid than for the other two materials. These findings are consistent with the results of this study, which also found polyglycolic acid to be the superior choice in terms of its mechanical behavior over time. While the actual tensile strengths reported are dissimilar from those reported in this study, both agree in the general direction, indicating polyglycolic acid was the material with the highest performance. The difference in values may be due to differences in methodology between the studies with regard to the immersion medium and conditions of study. The sutures in the study by Khiste et al. [4] were submerged in artificial saliva and placed in an incubator at 37°C, simulating the oral cavity. This difference may have contributed to the degradation of the materials, thereby affecting the mechanical strength of the materials being measured.

In the study of Manfredini et al. [11], 24 types of suture threads of 3/0, 4/0, and 5/0 caliber were compared, among them silk and polyglycolic acid. In the results they mention that silk and polyglycolic acid showed higher tensile strength values in the 4/0 and 5/0 caliber groups, respectively. In this study, the values obtained in the results were not similar either, since it was immersed in artificial saliva with a pH of 7.4; therefore, we could conclude that, with alkaline substances, the resistance to traction is affected more than with acid substances. Based on the results obtained in this research, it can be concluded that polyglycolic acid represents a highly favorable alternative as a suture material due to its superior tensile strength and its remarkable mechanical stability over time. These are characteristics of great importance to him in every surgical wound, because they aid in the preservation of the integrity of the wound at the crucial period in the healing process. The increased resistance of the thread provides consequent closure of soft tissues, gradually favored and not compromised by the presence of the thread, which makes for a more even and wholesome recovery with less tendency to complications than threads of lesser strength are capable of promoting [12]. Thus, service to the patient might be enhanced were an optimum clinical result obtainable with polyglycolic acid.

Like in the present study, several authors have reported that the tensile strength of the suture threads decreases over time of immersion in aggressive media. Anyusha et al. [13] demonstrated that, under conditions of exposure to acidic solutions, both types of sutures, those that are absorbable and those that are not, become mechanically degraded over a period closely resembling that which has been evaluated by us (up to 14 days).

Although there was no significant difference between the influence of Camu Camu and Tumbo juices in the investigation, studies such as that of Anushya et al. [13] Have explored the influence of the pH and other chemical components of the medium on suture degradation. Abellan et al. [10] concluded that the acidity and organic composition of the medium can accelerate the degradation process, although in their study no marked differences were found between different liquids with acidic characteristics. This similarity with my results suggests that the variability in the chemical composition among the juices evaluated was not sufficient to generate significant differences in suture degradation. The importance of these results lies in their clinical application in periodontal and implant surgeries, in guiding clinicians in the appropriate selection of the type of suture thread according to the surgery they wish to perform14 and in suggesting that fruit juices should be consumed with caution when performing these types of surgeries, where it is important to keep the tissues together [14–18].

The limitations of this study were that, as an *in vitro* study, the data obtained on the tensile strength of the suture threads could vary clinically. Saliva helps regulate the pH of the oral cavity due to its buffering effect. Therefore, the suture thread would not be under a constant acidity level. The temperature of the oral cavity could also have an influence, as the specimens were kept at 21°C. We suggest that future studies assess the behavior of suture materials using saliva, variable temperature, or masticatory mechanical loads, or use variable oral pH in the studies to improve the accuracy of the results. Using those conditions would render results useful in the daily practice of the dentist, enabling the understanding of how those materials behave in the oral environment and assisting in the decision of which of the types of thread is the most proper for each surgical procedure, in the establishment of a healing pattern, and in the durability of sutures for the patient.

Some suture brands could present differences in relation to their tensile strength towards the acid environment. These differences are due to the manufacturing process, composition of the polymers and coating technologies, and possibly some specific characteristics of each brand. This could indicate important changes in their resistance to the acid medium, because immersing them in the camu camu or tumbo juices may produce different degradation rates depending on the established brand. A test that would be useful would be to evaluate which manufacturer produced the highest tensile strength compared to the others through immersion in the juices of our study.

Another significant limitation of this study was that it did not strictly control the temperature of the juices or suture specimens. Both the juice and suture specimens were retained at 21°C, which is significantly lower than the physiological temperature of the oral cavity (i.e., ~37°C). The tensile strength assays were completed under “cooler” conditions, which therefore may have slowed the degradation of the suture, and the findings may not be as representative of the results in clinical use. The mechanical stability in this study may therefore over‐predict the durability of absorbable sutures in vivo. Research studies in the future should consider using a temperature‐controlled environment to provide more clinically relevant results. The absence of a neutral control medium (for example, saline or artificial saliva) is one of the limitations of this research study. As a result, although the differences between nylon sutures and polyglycolic acid sutures due to acidity were compared in this study, the specific effects of acidity were not measured. Future studies need to include neutral controls in order to increase the reproducibility and validity of pH’s role in suture degradation.

However, the experimental approach allowed the observation of the progressive behavior of the materials under conditions that simulate, in part, the oral environment, thus providing valuable information for the appropriate selection of suture threads based on their mechanical performance over time and their interaction with different natural products [19–21].

## 5. Conclusions

This study demonstrates how the tensile strength of nylon and polyglycolic acid sutures decreases over time, highlighting the differences between the two materials and the impact of the juices used. Polyglycolic acid stands out for its durability, remaining stronger than nylon in all evaluations. Camu and Tumbo juices possessed a similar effect on degradation of the sutures, but the effect of pH on loss of strength was determined to be significant, with a loss of strength noted for each change of 1 pH unit. This work invites thought on the determination of a suitable, obvious chemical environment in which absorbable sutures can be used. We may improve the choice of material and result in practice work of the various surgical sutures or intestinal clips.

## Author Contributions

**Tania Mamani-Salinas**, **Daniel Alvitez-Temoche**, **Julia Medina**, **Cesar Mauricio-Vilchez**, **Oscar Sotomayor**, **Frank Mayta-Tovalino**, **and Fran Espinoza-Carhuancho**: concept and design of study, drafting, revision. **Fran Espinoza-Carhuancho**, **Tania Mamani-Salinas**, **and Frank Mayta-Tovalino**: acquisition of data, analysis, interpretation. **Tania Mamani-Salinas**, **Julia Medina**, **Oscar Sotomayor**, **Daniel Alvitez-Temoche**, **and Frank Mayta-Tovalino**: acquisition of data, interpretation, drafting.

## Funding

The authors received no specific funding for this work.

## Disclosure

All authors have given approval of the version of the article to be published.

## Ethics Statement

The authors have nothing to report.

## Conflicts of Interest

The authors declare no conflicts of interest.

## Data Availability

The datasets used and/or analyzed during the current study are available from the corresponding author upon reasonable request.
